# Clinical significances of p27 in digestive tract cancers: a comprehensive analysis on immunohistochemistry staining, published literatures, microarray and RNA-seq data

**DOI:** 10.18632/oncotarget.24316

**Published:** 2018-01-23

**Authors:** Dan-Dan Xiong, Rong-Quan He, Ai-Hua Lan, Wen-Jie Chen, Yi-Huan Luo, Zhi-Hua Ye, Jie Ma, Gang Chen, Yi-Wu Dang

**Affiliations:** ^1^ Department of Pathology, First Affiliated Hospital of Guangxi Medical University Nanning, Guangxi Zhuang Autonomous Region 530021, China; ^2^ Department of Medical Oncology, First Affiliated Hospital of Guangxi Medical University Nanning, Guangxi Zhuang Autonomous Region 530021, China

**Keywords:** digestive tract cancers, p27, immunohistochemistry, prognoses, clinical significances

## Abstract

In the present study, we conducted a comprehensive analysis on the clinical roles of p27 protein and p27 gene in digestive tract cancers (DTCs). First, we performed immunohistochemistry staining and found that p27 protein was down-regulated in DTCs. Then we collected 62 publications and calculated the combined hazard ratios (HRs), odds ratios (ORs) and 95% confidence intervals (95% CIs) to clarify the relationships of p27 protein expression with prognoses and clinicopathological parameters. The overall HRs indicated that the down-regulated p27 protein was an independent prognostic biomarker for overall survival (HR: 1.58, 95% CI: 1.38–1.81, *P* < 0.0001) but not for disease–free survival and cancer–specific survival. The combined ORs indicated that a low expression of p27 protein was positively related to lymph node metastasis (OR: 2.15, 95% CI: 1.57–2.96, *P* < 0.0001), distant metastasis (OR: 2.02, 95% CI: 1.12–3.63, *P =* 0.019) and pathology grading (OR: 2.14, 95% CI: 1.75–2.62, *P* < 0.0001). Additionally, 60 DTCs-related microarray and RNA-seq datasets were obtained to investigate the expression level and clinical value of p27 gene in DTCs patients. We found that the expression level of p27 gene in DTCs was similar to that in normal controls. And no significant associations of p27 gene expression with prognoses and clinicopathological factors were observed. In conclusion, according to our results, it was p27 protein, but not p27 gene, that can function as an effective biomarker to predict the clinical outcome in patients with DTCs. The down-regulation of p27 protein in DTCs may not result from the altered expression of p27 gene.

## INTRODUCTION

Digestive tract cancers (DTCs) are primary cancer burdens worldwide and include esophageal cancer (ESCA), gastric cancer (GC) and colorectal cancer (CRC), which are the ninth, sixth and third most common cancers in the world, respectively [1]. The incidence of DTCs has increased due to aging and growing populations and to increasing smoking and obesity rates [2, 3]. Although the diagnosis and treatment of DTCs have been improved for the past decades, the long-term survival rate for DTCs patients is still dismal and the cancer-related mortality rate is still high. Therefore, probing effective biomarkers for early diagnosis and clinical outcome prediction is imperative, which is helpful in better understanding the development of DTCs and further improving the survival outcome in patients with DTCs.

Protein p27, also known as CDKN1B (cyclin-dependent kinase inhibitor 1B), is a tumor suppressor protein of the CIP/KIP family and is encoded by p27 gene that located on chromosome 12p13. Protein p27 has been demonstrated to be a vital regulator of cell cycle progression via restraining cyclin E- and cyclin A-CDK2 complexes and preventing cells into S phase of the cell cycle [4]. Increasing researches have shown that p27 protein functions as a tumor suppressor to regulate both cell proliferation and tissue expansion in various malignancies. A growing number of studies have reported that p27 protein expression could be used to predict clinical outcomes in various malignant tumors, such as breast cancer [5], lung cancer [6] and ovarian cancer [7]. The clinical and prognostic values of p27 protein expression in patients with DTCs have also been investigated, with different studies reporting conflicting results; most studies have showed that a low p27 expression is related to a poor clinical outcome in DTCs patients [8, 9]. However, some contradictory results were also achieved [10, 11]. Thus. validation by independent studies working on different case series is urgently required before p27 protein can be served as a biomarker in clinic for diagnosis and clinical outcome prediction in patients DTCs. Therefore, we collected all available published literatures those referred to the clinical and prognostic significances of p27 protein in DTCs and performed immunohistochemistry (IHC) staining using clinical tissues from in-house DTCs patients to clarify the expression and clinical value of p27 protein in DTCs. Additionally, DTCs-related microarray and RNA-seq datasets were also obtained from the Gene Expression Omnibus (GEO), Oncomine, ArrayExpress and The Cancer Genome Atlas (TCGA) to further investigate the expression level and clinical significances of p27 gene in DTCs patients.

## RESULTS

**Figure 1:**
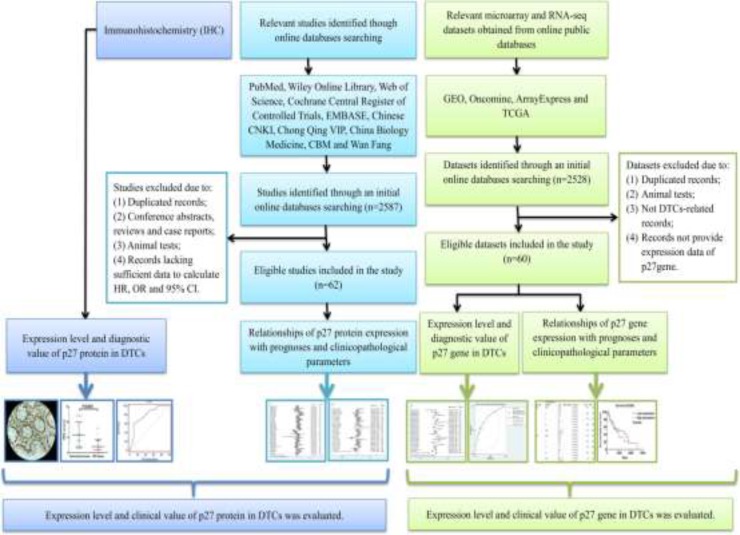
Flow chart of the overall design of the current study.

In the present study, IHC staining was conducted and relevant published literatures, microarray and RNA-seq datasets were collected to explore the expression patterns and clinical significances of p27 gene and protein in DTCs. The overall design of the current study is presented in Figure 1.

### Expression level of p27 protein in DTCs based on IHC staining

**Figure 2:**
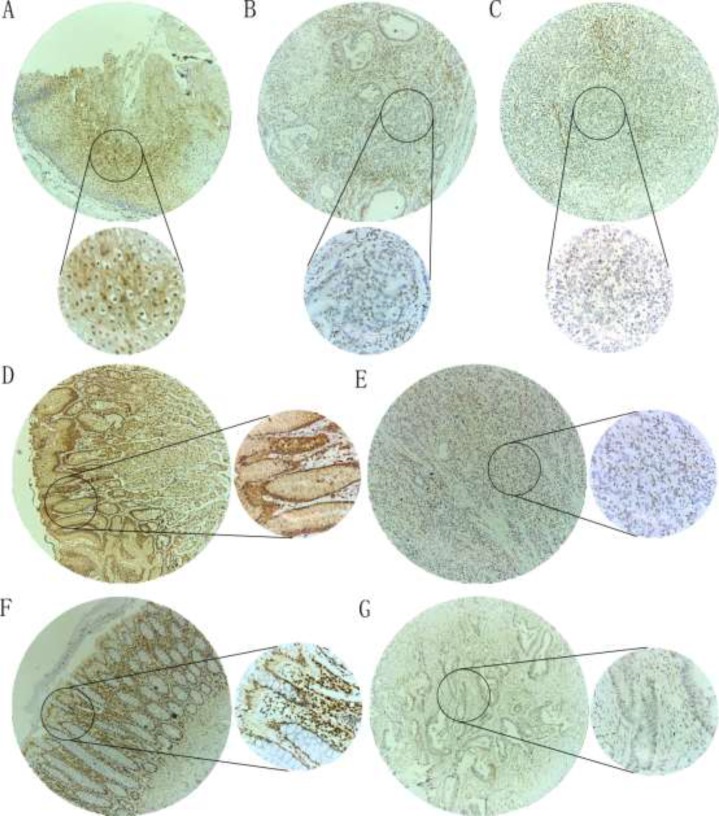
IHC staining of p27 protein in DTCs and adjacent non-tumorous tissues Cytoplasmic and/or nuclear staining was observed for p27 protein. (**A**) A paracancerous esophagus tissue showed a high p27 protein expression (magnification: ×100 (upper) and ×400 (lower)); (**B**) A esophagus adenocarcinoma tissue showed a low p27 protein expression (magnification: ×100 (upper) and ×400 (lower)); (**C**) A esophagus squamous cell carcinoma tissue showed a low p27 protein expression (magnification: ×100 (upper) and ×400 (lower)); (**D**) A paracancerous gastric tissue showed a high p27 protein expression (magnification: ×100 (left) and ×400 (right)); (**E**) A gastric cancer tissue showed a low p27 protein expression (magnification: ×100 (left) and ×400 (right)); (**F**) A paracancerous colorectal tissue showed a high p27 protein expression (magnification: ×100 (left) and ×400 (right)); (**G**) A colorectal cancer tissue showed a low p27 protein expression (magnification: ×100 (left) and ×400 (right)).

**Figure 3:**
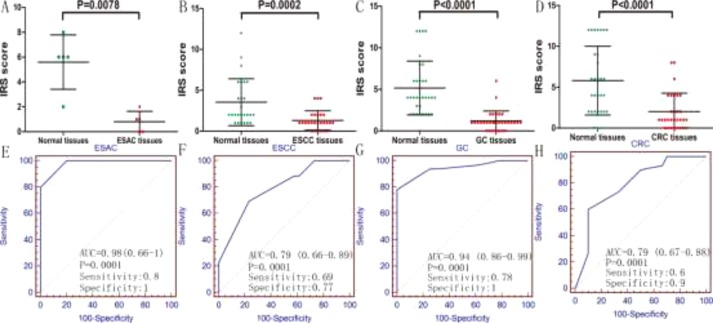
Expression level and diagnostic value of p27 protein in ESAC (**A** and **E**), ESCC (**B** and **F**), GC (**C** and **G**) and CRC (**D** and **H**).

Positive rate of p27 protein expression was 0% (0/5) in esophageal adenocarcinoma (ESAC) and 80% (4/5) in paired normal esophagus tissues, 0% (0/26) in esophageal squamous carcinoma (ESCC) and 26.9% (7/26) in paired normal esophagus tissues, 3% (1/32) in GC and 37.5% (12/32) in paired normal gastric tissues, 10% (3/30) in CRC and 50% (15/30) in paired normal colorectal cancer (Figure 2). The scatter diagram and paired *T*-test were utilized to compare the expression of p27 protein in DTCs tissues and adjacent non-cancerous tissues, and the results indicated that the expression level of p27 protein was lower in ESAC (*P* = 0.0078; Figure 3A), ESCC (*P* = 0.0002; Figure 3B), GC (*P* < 0.0001; Figure 3C) and CRC (*P* < 0.0001; Figure 3D) tissues than in paired non-tumorous tissues. The receiver operating characteristic (ROC) curve was generated to estimate the diagnostic capability of p27 protein in DTCs. The area under the curve (AUC) values of p27 protein in ESAC, ESCC, GC and CRC were 0.98 (95% CI: 0.66–1, *P* = 0.0001; sensitivity: 0.8; specificity: 1; Figure 3E), 0.79 (95% CI: 0.66–0.89, *P* = 0.0001; sensitivity: 0.69; specificity: 0.77; Figure 3F), 0.94 (CI: 0.86–0.99, *P* = 0.0001; sensitivity: 0.78; specificity: 1; Figure 3G) and 0.79 (95% CI: 0.67–0.88, *P* = 0.0001; sensitivity: 0.6; specificity: 0.9; Figure 3H), respectively.

Additionally, we also validated the expression level of p27 protein based on The Human Protein Atlas database (http://www.proteinatlas.org/), and the results are displayed in Supplementary Figure 1.

### Clinical significances of p27 protein in DTCs based on published literatures

#### Result of literature search

As shown in Figure 1, a total of 62 publications (including 11 for ESCC, 26 GC for and 25 for CRC) with 9244 cases were involved in the present study. All of the literatures were published from 1997 to 2016. The expression of p27 protein was only assessed by regular IHC staining. The positive standards were inconsistent due to the use of different antibodies from different manufacturers. The follow-up times ranged from 29 to 240 months, the cut-off values ranged from 0.03 to 0.75, and the sample sizes ranged from 23 to 1062. Among the 62 studies, 54 studies [8, 10–62] followed survival, and 37 studies [8, 13, 15–20, 24, 28–30, 36, 39, 40, 44–47, 49–51, 54, 55, 57–61, 63–70] investigated the relationships of p27 protein expression with lymph node metastasis, distant metastasis and pathology grading. Among the 54 survival-related studies, 17 articles provided HRs and 95% CIs directly, while 33 articles only provided Kaplan–Meier survival curves. The basic information and quality scores of the 62 eligible studies are summarized in Supplementary Table 2.

### Relationships of p27 protein expression with prognoses and clinicopathological parameters

**Figure 4:**
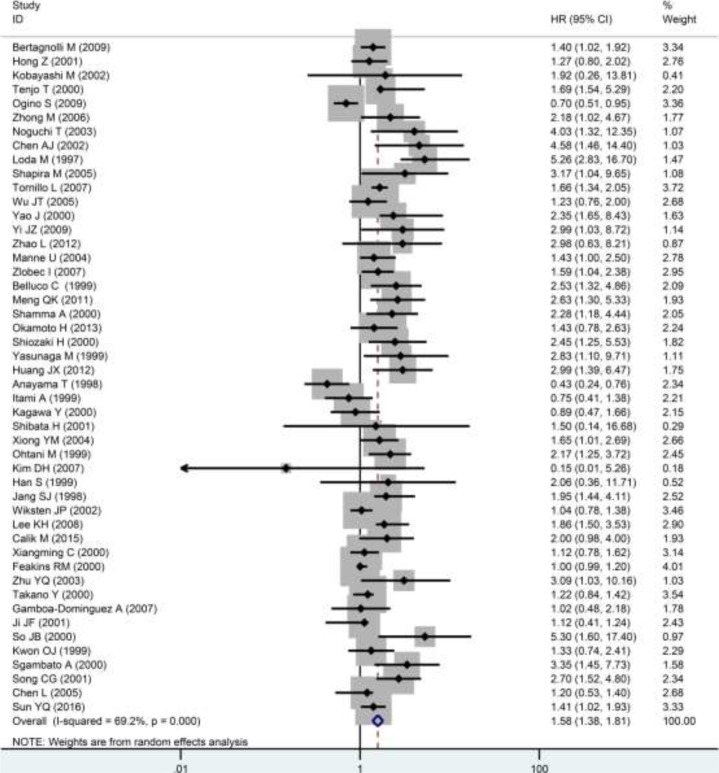
Forest plot of the pooled HR for OS HR>1 indicates a poor OS for the group with a decreased p27 protein expression.

**Figure 5:**
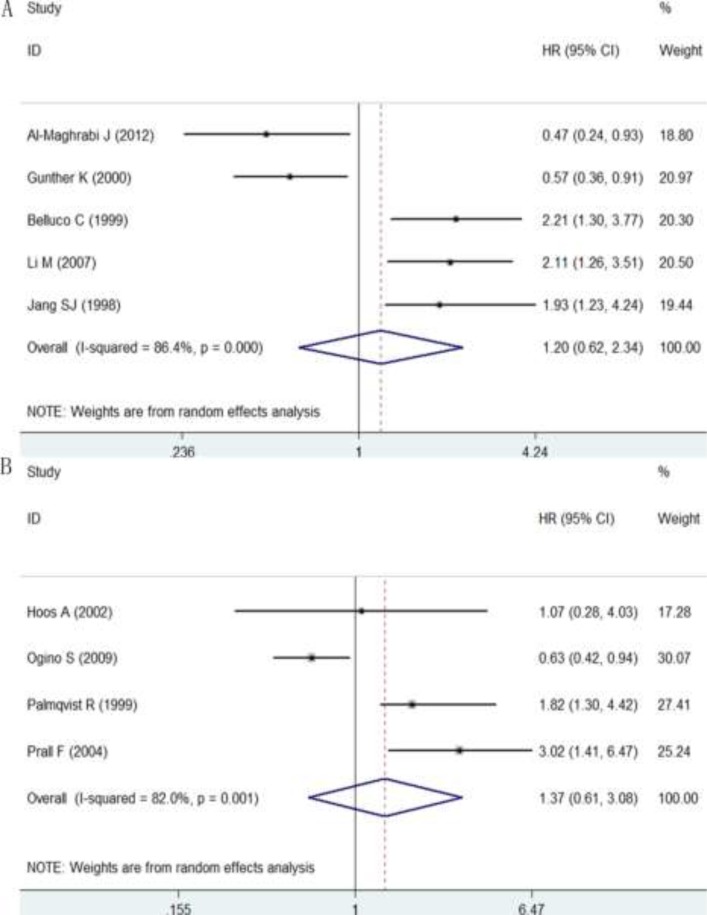
Forest plot of the pooled HR for DFS (**A**) and CSS (**B**).

A total of 48 reports with 8537 cases reported the correlation between p27 protein expression and overall survival (OS). The pooled hazard ratio (HR) from a random-effects model indicated that a low expression of p27 protein predicted a poor OS (HR: 1.58, 95% CI: 1.38–1.81, *P* < 0.0001; I^2^ = 69.2%, *P* < 0.0001; Figure 4; Table 1) in patients with DTCs. Then subgroup analyses according to cancer types, statistical methods and cut-off values were conducted. Results of subgroup analysis on cancer types showed that a down-regulated p27 protein indicated an unfavorable OS in patients with GC (HR: 1.47, 95% CI: 1.23–1.76, *P* < 0.0001; Supplementary Figure 2; Table 1) and CRC (HR: 1.81, 95% CI: 1.44–2.27, *P* < 0.0001; Supplementary Figure 2; Table 1), but not in patients with ESCC (HR: 1.41 95% CI: 0.92–2.14, *P* = 0.114; Supplementary Figure 2; Table 1). Subgroup analysis on statistical methods were also performed, and the HRs in multivariate analysis, univariate analysis and survival curve groups were 1.73 (95% CI: 1.27–2.34, *P* < 0.0001; Supplementary Figure 3; Table 1), 3.07 (95% CI: 1.83–5.14, *P* < 0.0001; Supplementary Figure 3; Table 1) and 1.46 (95% CI: 1.28–1.68, *P* < 0.0001; Supplementary Figure 3; Table 1), respectively. Results of subgroup analysis on cut-off values demonstrated that a reduced p27 protein expression was remarkably linked with a worse OS regardless of high (>0.25) or low (≤0.25) cut-off values, and the HRs were 1.63 (95% CI: 1.33 -2.0, *P* < 0.0001) and 1.46 (95% CI: 1.21–1.76, *P* < 0.0001), respectively (Supplementary Figure 4, Table 1). Additionally, a total of 5 studies with 564 patients investigated the relationship between p27 protein expression with disease-free survival (DFS) and 4 studies with 960 patients explored the association of p27 protein expression with cancer-specific survival (CSS). However, no statistically significant difference between p27 protein expression and DFS (HR: 1.20 95% CI: 0.62–2.34, *P* = 0.594; I^2^ = 86.4%, *P* < 0.0001; Figure 5A; Table 1) or CSS (HR: 1.37 95% CI: 0.61–3.06, *P* = 0.445; I^2^ = 82%, *P* = 0.001; Figure 5B; Table 1) was observed.

**Table 1:** The results of meta-analysis for OS, DFS and CSS

Survival outcome	Group	Number of studies	Number of patients	HR (95% CI)	*P* value	Heterogeneity test		Publication bias	
						*I*^2^ (%)	*P* value	Begg’s *P*	Egger’s *P*
**OS**		48	7853	1.58 (1.38–1.81)	<0.0001	69.2	<0.0001	0.93	0.844
	**Cancer type**								
	ESCC	10	773	1.41 (0.92–2.14)	0.114	71.6	<0.0001		
	GC	19	2386	1.47 (1.23–1.76)	<0.0001	66.1	<0.0001		
	CRC	19	4694	1.81 (1.44–2.27)	<0.0001	64.9	<0.0001		
	**Statistical methods**								
	Multivariate analysis	13	2374	1.73 (1.27–2.34)	<0.0001	80.3	<0.0001		
	Univariate analysis	2	153	3.07 (1.83–5.14)	<0.0001	0	0.318		
	Survival curve	33	5326	1.46 (1.28–1.68)	<0.0001	48.4	0.001		
	**Cut-off value**								
	≤0.25	20	3551	1.46 (1.21–1.76)	<0.0001	66.5	<0.0001		
	>0.25	25	3849	1.63 (1.33–2.0)	<0.0001	64.6	<0.0001		
DFS		5	564	1.20 (0.62–2.34)	0.594	86.4	<0.0001	None	None
CSS		4	960	1.37 (0.61–3.06)	0.445	82	0.001	None	None

**Figure 6:**
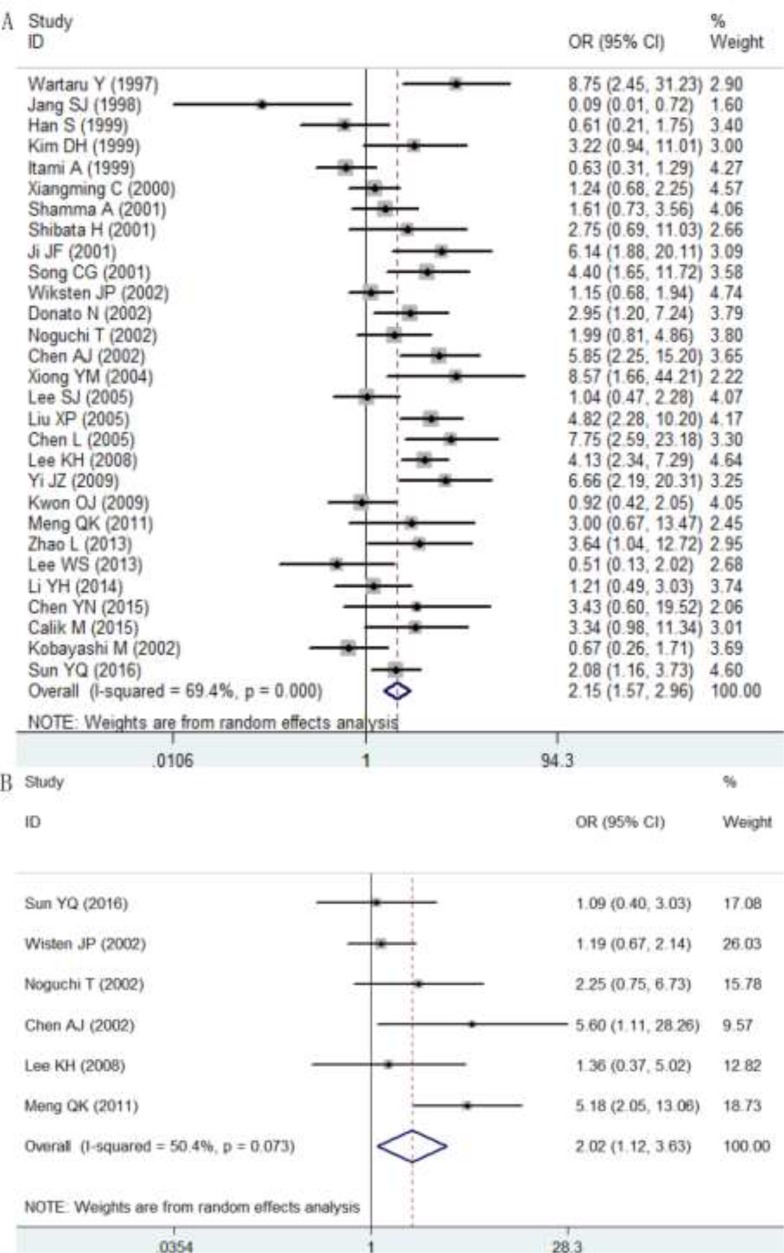
Relationships of p27 protein expression with lymph node metastasis (**A**) and distant metastasis (**B**). OR >1 indicates that a decreased p27 expression was positively correlated with lymph node metastasis and distant metastasis.

**Figure 7:**
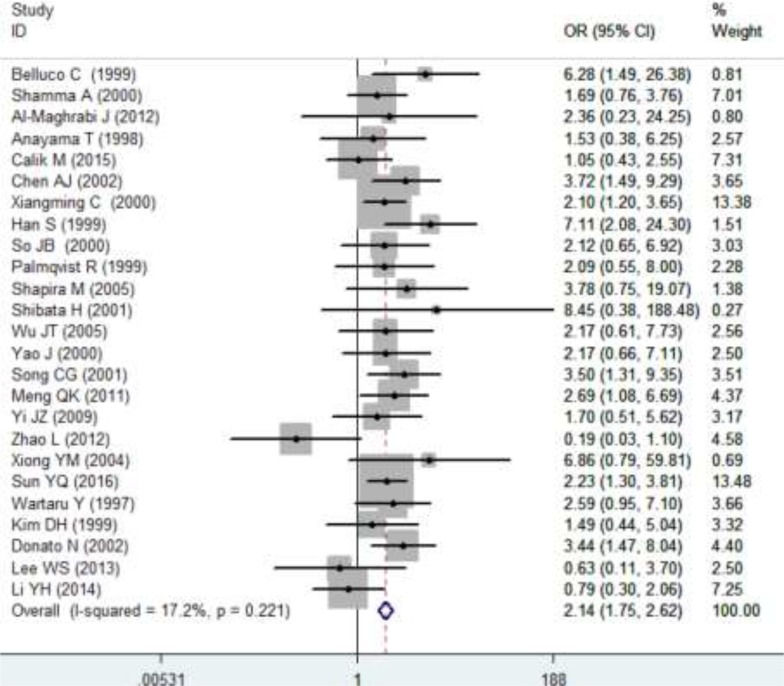
Relationships between p27 protein expression and pathology grading OR>1 indicates that a decreased p27 expression was positively correlated with pathology grading.

Furthermore, the correlations of p27 protein expression with lymph node metastasis, distant metastasis and pathology grading were also assessed, and the results are concluded in Table 2. In total, 29 studies investigated the correlation between p27 protein expression and lymph node metastasis, 6 studies reported the relationship between p27 protein expression and distant metastasis and 25 studies evaluated the association of p27 protein expression with pathology grading. The overall odd ratios (ORs) indicated that a decreased expression of p27 protein was positively related to lymph node metastasis (OR: 2.15, 95% CI: 1.57–2.96, *P* < 0.0001; I^2^ = 69.4%, *P* < 0.0001; Figure 6A), distant metastasis (OR: 2.02, 95% CI: 1.12–3.63, *P* = 0.019; I^2^ = 50.4%, *P* = 0.073; Figure 6B) and pathology grading (OR: 2.14, 95% CI: 1.75–2.62, *P* < 0.0001; I^2^ = 17.2%, *P* = 0.221; Figure 7) in patients with DTCs. A subgroup analysis on cancer types was conducted. In ESCC group, a low expression of p27 protein was linked with pathology grading (OR: 2.16, 95% CI: 1.15–4.05, *P* = 0.017) but not with lymph node metastasis (OR: 1.97, 95% CI: 0.83–4.69, *P* = 0.124). In GC group, correlations of p27 protein expression with lymph node metastasis (OR: 2.05, 95% CI: 1.38–3.04, *P* < 0.0001) and pathology grading (OR: 2.08, 95% CI: 1.62–2.69, *P* < 0.0001) were observed; while no significant associations between p27 protein expression and distant metastasis was found (OR: 1.19, 95% CI: 0.74–1.91, *P* = 0.463). In CRC group, a down-regulated p27 protein was positively correlated with lymph node metastasis (OR: 2.79, 95% CI: 1.33–5.88, *P* = 0.007; Figure 6A), distant metastasis (OR: 3.92, 95% CI: 2.05–7.49, *P* < 0.0001) and pathology grading (OR: 2.28, 95% CI: 1.55–3.36, *P* < 0.0001).

**Table 2:** The relationships of p27 protein expression with lymph node metastasis, distant metastasis and pathology grading

Clinicopathological features	Group	Number of studies	Number of patients	OR (95% CI)	*P* value	Heterogeneity test		Publication bias	
						*I*^2^ (%)	*P* value	Begg’s *P*	Egger’s *P*
Lymph node metastasis	Overall result	29	4208	2.15 (1.57–2.95)	<0.0001	69.4	<0.0001	0.586	0.611
	ESCC	5	462	1.97 (0.83–4.69)	0.124	65.4	0.021		
	GC	18	3100	2.05 (1.38–3.04)	<0.0001	72.6	<0.0001		
	CRC	6	646	2.79 (1.33–5.88)	0.007	64.8	0.014		
Distant metastasis	Overall result	6	921	2.02 (1.12–3.63)	0.019	50.4	0.073	None	None
	GC	3	694	1.19 (0.74–1.91)	0.463	0	0.967		
	CRC	3	247	3.92 (2.05–7.49)	<0.0001	0	0.467		
Pathology grading	Overall result	25	2344	2.14 (1.75–2.62)	<0.0001	17.2	0.221	0.907	0.911
	ESCC	4	270	2.16 (1.15–4.05)	0.017	0	0.489		
	GC	11	1139	2.08 (1.62–2.69)	<0.0001	32.3	0.141		
	CRC	10	935	2.28 (1.55–3.36)	<0.0001	21.8	0.242		

Abbreviations: SD: standard deviation.

Publication bias were also detected using Begg’s and Eeggr’s tests, and the results showed no publication bias in all situations (Table 1 and Table 2; Supplementary Figure 5).

### Expression level and clinical significances of p27 gene in DTCs based on microarray and RNA-seq datasets

#### Expression level and diagnostic performances of p27 gene in DTCs

A total of 60 mRNA microarray and RNA-seq datasets including 6 for ESAC, 9 for ESCC, 20 for GC and 25 for CRC were mined in the present study. The essential information of the 60 datasets is showed in Supplementary Table 2.

**Figure 8:**
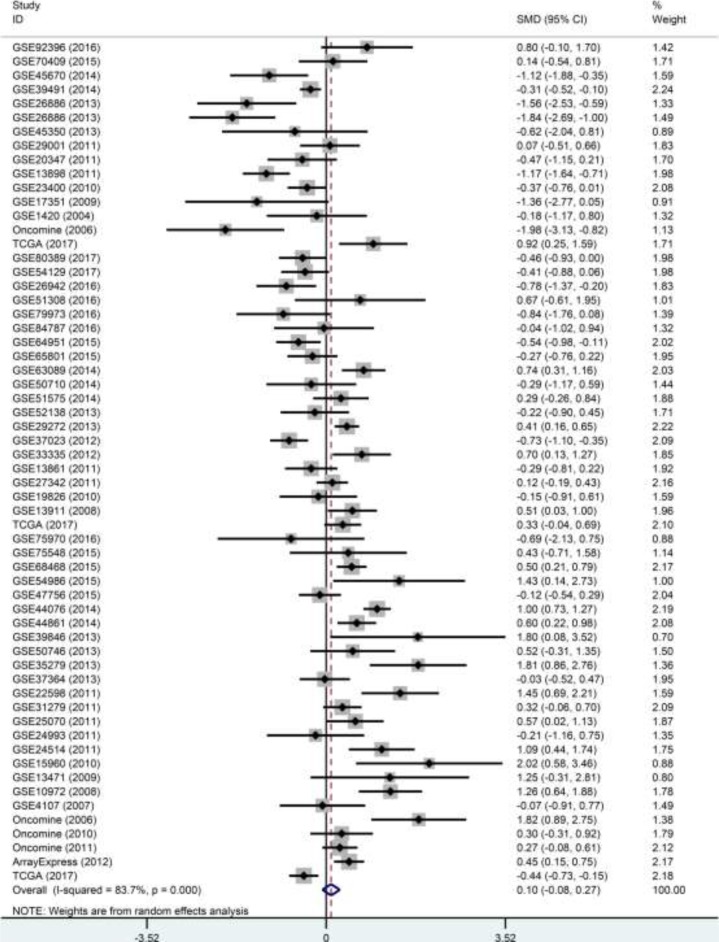
Forest plot of the 60 datasets evaluating p27 gene expression in DTCs (random-effects model) SMD >0 indicates that p27 gene was up-regulated in DTCs.

**Figure 9:**
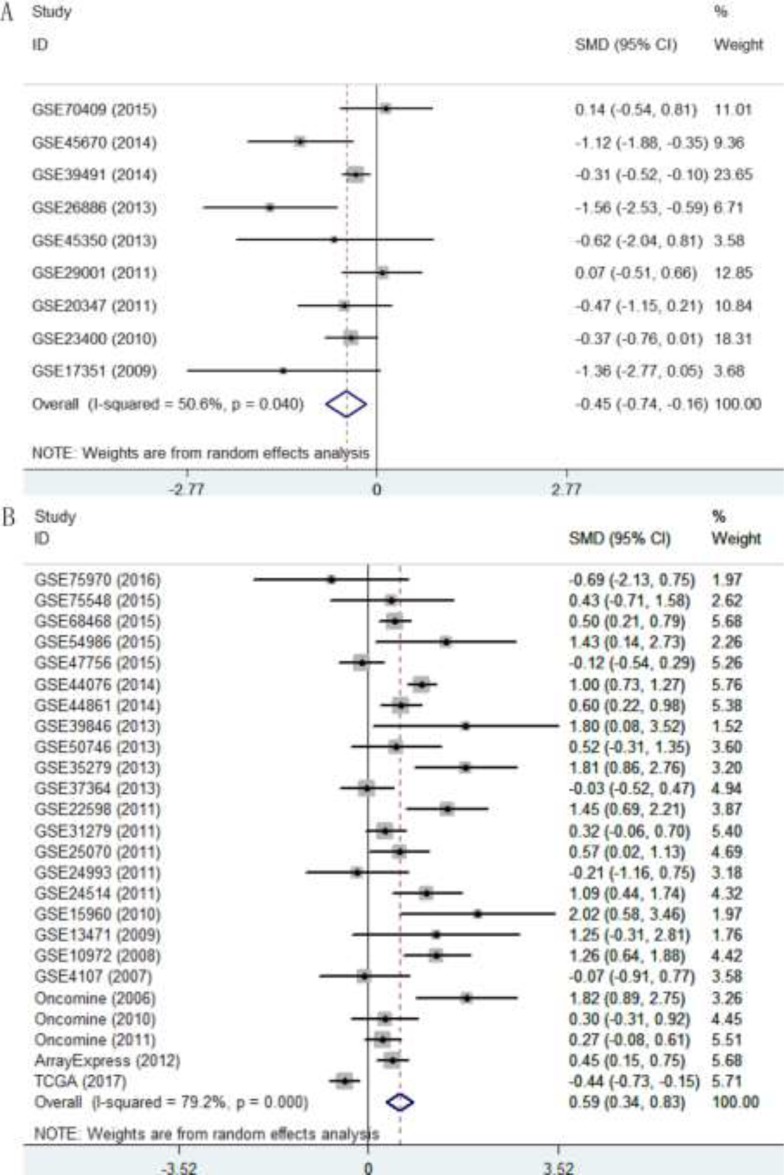
Forest plot evaluating p27 gene expression in ESCC and CRC (**A**) Expression level of p27 gene in ESCC. (**B**) Expression level of p27 gene in CRC.

**Figure 10:**
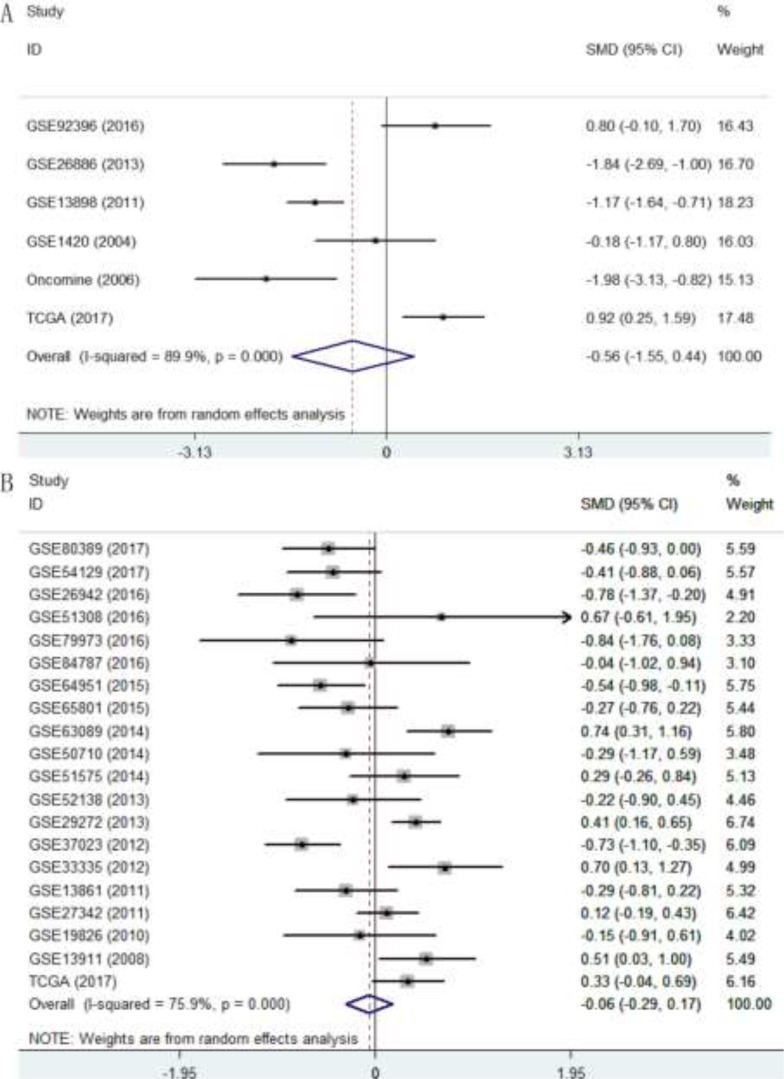
Forest plot evaluating p27 gene expression in ESAC and GC (**A**) Expression level of p27 gene in ESAC; (**B**) Expression level of p27 gene in GC.

To evaluate the expression level of p27 gene in DTCs, we integrated the 60 datasets. As shown in Table 3, the overall standard mean deviation (SMD) from a random-effects model was 0.1 (95% CI = -0.08∼0.27, *P* = 0.274; I^2^ = 83.7%, *P* < 0.0001; Figure 8), indicating that the expression level of p27 gene in DTCs was similar to that in normal controls. Then a subgroup analysis on cancer types was performed. The results showed that p27 gene was down-regulated in ESCC (SMD = -0.45, 95% CI = -0.74∼-0.16, *P* = 0.002; I^2^ = 50.6%, *P* = 0.04; Figure 9A) and up-regulated in CRC (SMD = 59, 95% CI = 0.34∼0.83, *P* < 0.0001; I^2^ = 79.2%, *P* < 0.0001; Figure 9B). While the pooled SMD for ESAC (SMD = -0.56, 95% CI = -1.55∼0.44 *P* = 0.273; I^2^ = 89.9%, *P* < 0.0001; Figure 10A) and GC (SMD = -0.06, 95% CI = -0.29∼0.17, *P* = 0.614; I^2^ = 75.9%, *P* < 0.0001; Figure 10B) suggested no statistically significant difference between cancerous and adjacent non-cancerous tissues. Additionally, the expression pattern of p27 gene in DTCs based on TCGA datasets were also displayed in the form of scatter plots and ROC curves (Supplementary Figure 6).

**Table 3:** The expression level and diagnostic performance of p27 gene in DTCs

Gorup	Number of datasets	SMD (95% CI)	*P* value	Heterogeneity		SROC curve		
				*I*^2^ (%)	*P* value	AUC (95% CI)	Sensitivity	Specificity
Overall result	60	0.10 (-0.08∼0.27)	0.274	83.7	<0.0001	0.79 (0.76–0.83)	0.60	0.84
ESAC	6	-0.56 (-1.55∼0.44)	0.273	89.9	<0.0001	0.82 (0.78–0.85)	0.76	0.86
ESCC	9	-0.45 (-0.74∼-0.16)	0.002	50.6	0.04	0.85 (0.82–0.88)	0.69	0.84
GC	20	-0.06 (-0.29∼0.17)	0.614	75.9	<0.0001	0.74 (0.70–0.78)	0.53	0.82
CRC	25	0.59 (0.34∼0.83)	<0.0001	79.2	<0.0001	0.81 (0.78–0.84)	0.61	0.84

Abbreviations: SROC curve: summary receiver operating characteristic curve; AUC: area under the curve.

Begg’s and Egger’s tests were conducted to estimate the publication bias among the 60 datasets, and the results demonstrated no publication bias (Begg’s *P* = 0.934, Egger’s *P* = 0.923; Supplementary Figure 7).

**Figure 11:**
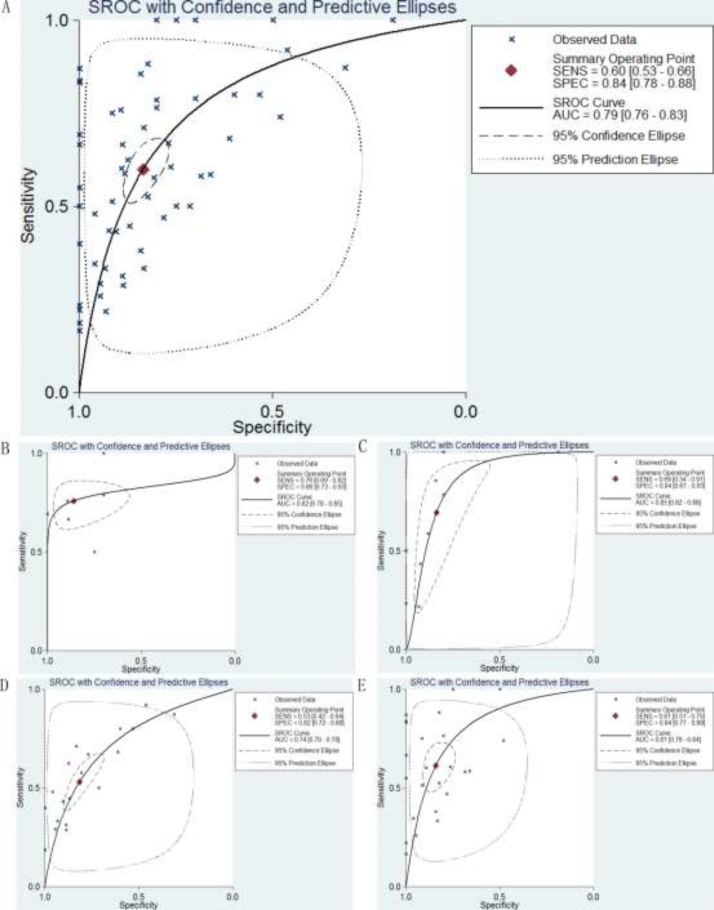
SROC curves for the identification of DTCs patients from normal controls using p27 gene expression (**A**) Diagnostic performance of p27 gene in DTCs; (**B**) Diagnostic performance of p27 gene in ESAC; (**C**) Diagnostic performance of p27 gene in ESCC; (**D**) Diagnostic performance of p27 gene in GC; (**E**) Diagnostic performance of p27 gene in CRC.

Furthermore, the summary receiver operating characteristic (SROC) curve was also generated to assess the ability of p27 in discriminating DTCs from normal controls. As shown in Table 3, the overall AUC of p27 gene in DTCs was 0.79 (95% CI: 0.76–0.83; Figure 11A), with sensitivity and specificity of 0.60 and 0.84, respectively. In addition, the AUC values of p27 gene in ESAC, ESCC, GC and CRC were 0.82 (CI: 0.78–0.85; sensitivity: 0.76; specificity: 0.86; Figure 11B), 0.85 (CI: 0.82–0.88; sensitivity: 0.69; specificity: 0.84; Figure 11C), 0.74 (CI: 0.70–0.78; sensitivity: 0.53; specificity: 0.82; Figure 11D) and 0.81 (CI: 0.78–0.84; sensitivity: 0.61; specificity: 0.84; Figure 11E), respectively.

### Clinical significances of p27 gene in DTCs from TCGA data

Expression data of p27 gene and corresponding clinicopathological information were downloaded from TCGA to explore the clinical value of p27 gene in DTCs. The correlations between p27 gene expression and several key clinicopathological parameters, including pathology grading, T stage, N stage, M stage and TNM stage, were assessed using Independent-samples *t*-test. The prognostic values of p27 gene in DTCs were assessed using Log-Rank test and Kaplan–Meier survival curve. However, according to our results, no significant associations of p27 gene expression with those key clinicopathological parameters and prognoses were observed (Supplementary Table 1 and Figure 8).

## DISCUSSION

The basic features of cancer are the disordered and uncontrolled cell cycle and the unlimited cell proliferation. As a member of cyclin-dependent kinase inhibitor family, p27 protein acts as a negative controller of cellular proliferation through inducing G1 arrest of the cell cycle, and thus inhibits the occurrence and development of malignant tumors [71–74]. A large number of researches have showed that the expression level of protein p27 in cancerous tissues is lower than that in normal controls, indicating that p27 protein may function as a tumor suppressor in malignancies. In our present study, we also detected the expression pattern of p27 protein in DTCs and adjacent non-cancerous tissues using IHC. Our results showed that p27 protein was down-regulated in ESAC, ESCC, GC and CRC, which are consistent with preceding researches [14, 25, 49, 75]. Then we further investigated the diagnostic ability of p27 protein in DTCs, and the results suggested that p27 protein could differentiate patients with DTCs from normal subjects to a degree. However, the results should be interpreted with caution since the number of the included in-house specimens is small. Therefore, further investigations with larger sample scales are necessary to verify our conclusion.

A study conducted by See et al [76] show that an increase in the number of chromatid breaks in protein p27-absence cells leads to a decrease in chromosome stability and ultimately a poor prognosis in cancer patients. Previous studies have also demonstrated that cancer patients with down-regulation of p27 protein are at high risk for poor clinical outcome [5, 7, 77, 78]. However, the clinical and prognostic value of protein p27 in DTCs has not been systematically investigated as of yet. Therefore, we collected all available relevant published literatures to synthetically analyze the relationships of p27 protein expression with prognoses and clinical parameters. A total of 54 publications were included to estimate the prognostic role of p27 protein in DTCs. The overall results indicated that the loss of p27 protein expression was an independent prognostic biomarker for OS but not for DFS and CSS in patients with DTCs. On the one hand, reports those investigated OS usually contained patient with longer follow-up time and included more cases to obtain statistical results on patient prognosis than those investigated DFS and CSS, and thus yielded stronger data. On the other hand, the number of included studies reporting DFS and CSS were only five and four, respectively. Thus, further studies with more samples are necessary to determine the correlations of p27 protein expression with DFS and CSS. Additionally, stratified analysis was also undertaken, and the results suggested that a decreased p27 protein expression in DTCs is predictive for OS independent of statistical method and cut-off value. However, subgroup analysis on cancer types showed that a down-regulated p27 protein predicted an unfavorable OS in GC and CRC but not in ESCC. A possible explanation is that the histologic type of ESCC is different from that of GC and CRC: CRC and GC are predominantly adenocarcinoma, while ESCC originates from squamous cells. The contributing manner of cell-cycle regulator p27 protein in ESCC is probably different from that in adenocarcinoma [17, 31, 79].

As well known, cancer patients with lymph node or distant metastasis often encounter poor survival outcome. Meanwhile, high grade of carcinoma pathology usually means poor tumor differentiation and prognosis. Therefore, digging an effective biomarker to predict cancer progression and identify high-risk patients, thereby optimizing individual treatment management and improving the prognosis of DTCs patients is imperative. According to our results, a reduced p27 protein expression in patients with DTCs is closely correlated with positive lymph node and distant metastases as well as poor tumor differentiation. Detection of p27 protein in patients with DTCs may be helpful in distinguishing high-risk patients and guiding the determination of individualized treatment protocols. Further and stricter clinical researches are needed to verify the conclusion.

Protein p27 is encoded by gene p27, while the cellular abundance of p27 protein is mainly regulated at the post-transcriptional level and the regulation is affected by a lot of factors. Accumulated evidences have demonstrated that phosphorylation induced by PKB/Akt [80, 81] or CyclinD1 [82] dominates the expression and stability of p27 protein. A study conducted by Loda et al [31] demonstrate that tumors with decreased p27 protein expression show increased proteolytic activity specific for protein p27, indicating that the down-regulated p27 protein may be due to the enhancive proteasome-induced degradation rather than the altered expression of p27 gene. To clarify the correlations between p27 protein and p27 gene in DTCs, we collected 61 microarray and RNA-seq datasets those contained the expression data of p27 gene. The overall result showed that the expression level of p27 gene in DTCs is similar to that in normal controls. Then a subgroup analysis by cancer types was also carried out, and the results indicated that p27 gene was down-regulated in ESCC but up-regulated in CRC. While in ESAC and GC, no altered expression of p27 gene was observed. Additionally, we also investigated the clinical and prognostic value of p27 gene in DTCs, and the results presented that no relationships of p27 gene expression with clinicopathological parameters and prognoses were found. Our findings suggested that there was no correlation between p27 protein and p27 gene expression in DTCs, which was in accordance with preceding reports conducted by Hunter et al [83] and Li et al [84].

Although our results revealed that p27 protein was down-regulated in DTCs and the reduced expression of p27 protein was closely related to poor clinical outcome, the conclusions should be interpreted cautiously since there were several limitations in our study. First, the combined HR used in our study induced a key bias due to the use of different statistical methods in the included studies, including multivariate analysis, univariate analysis and survival curves. The HRs acquired from multivariate analyses were more accurate than those from univariate analyses because intermixed factors were included in the multivariate analyses. Additionally, in several reports, the HRs were not provided directly and had to be calculated from the Kaplan–Meier survival curves, which may result in inevitable errors and decrease the accuracy of the results. Second, extensive significant heterogeneities were observed in our study. The noticeable heterogeneities may result from the differences in the following characteristics of included records: sample sizes, detection methods and platforms, primary antibodies and dilutions, cut-off values, preoperative treatments, cancer types, case selection criteria, population differences in regions, gender ratios, tumor stages, and status of lymph node and distant metastases. Third, the length of follow-up times in the included trials ranged from 29 months to 240 months, and the number of participants varied from 23 to 1062. We did not exclude studies with limited follow-up times or small patient numbers. However, this may introduced biases because the limited durations and study sizes are likely to produce unreliable data. Fourth, our study included fully published studies in English or Chinese and excluded studies written with other languages, which may introduced potential language bias. Additionally, researches with positive results are more likely to be published. Therefore, we should not ignore the potential bias in the present study even though the results from Begg’s and Egger’s tests showed no publication bias.

To sum up, our results suggested p27 protein was down-regulated in DTCs and a decreased expression of p27 protein predicted an unfavorable clinical outcome. Detecting p27 protein in patients with DTCs may be helpful in distinguishing high-risk patients and guiding the determination of individualized treatment protocols. More interesting, we also discovered that the expression level and clinical significances of p27 protein were inconsistent with those of p27 gene, indicating that the down-regulation of p27 protein did not result from the altered expression of p27 gene. Further well-designed studies are warranted to validate our conclusions.

## MATERIALS AND METHODS

### Immunohistochemistry

The expression level of p27 protein was detected by IHC method in 26 ESCC, 5 ESAC, 32 GC, 30 CRC and their adjacent normal tissues. All samples were obtained from patients who underwent surgery without neoadjuvant treatment at Guangxi Medical University, People’s Republic of China from May 2015 to April 2017. The formalin-fixed paraffin-embedded tissues were cut into 4-μm thick sections and deparaffinized. Antigen retrieval was performed in EDTA buffer with pressure cooking at 100°C for 4 minutes. Endogenous peroxidase was inactive in 3% hydrogen peroxide for 15 minutes. Then the sections were incubated with rabbit monoclonal anti-p27 KIP antibody (1:100 dilution, Abcam, USA) for 1 h at 37°C. The rest procedure was conducted according to the manufacturer’s instruction. Two pathologists (Gang Chen and Yi-wu Dang) read each immunostained tissue under microscopy and scored it independently according to the following two criteria: (1) the staining intensity was determined as 0 (no staining), 1 (weak staining), 2 (moderate staining) and 3 (strong staining); (2) the staining percentage of tumor cells was scored as 0 (<5%), 1 (5%–25%), 2 (26%–50%), 3 (51%–75%), 4 (76%–100%). The positive percentage of cytoplasm and/or nuclei of cells were calculated in more than 1000 cells of five successive and representative high power fields (×400 magnification microscope). The immunoreactive score (IRS) was applied to determine the final staining score by multiplication of the intensity score and the distribution score. Patients were divided into p27 positive group (IRS ≥ 6) and p27 negative group (IRS < 6). Then the scatter diagram was generated using GraphPad Prism 5 (La Jolla, CA, USA) and the SROC curve analysis was performed using Stata12.0 (Stata Corporation, College Station, TX, USA) based on the IRS scores to evaluate the expression pattern and diagnostic capability of p27 protein in DTCs.

### Data mining

#### Data acquisition from published literature

A comprehensive and systematic literature search was conducted in 10 online databases, including Wiley Online Library, Web of Science, Cochrane Central Register of Controlled Trials, EMBASE, PubMed, Chinese CNKI, Chong Qing VIP, China Biology Medicine disc, CBM and Wan Fang (with an upper date limit of August 15, 2017), using the following strategy: (malignan* OR cancer OR tumor OR tumour OR neoplas* OR carcinoma OR adenocarcinoma) AND (digestive OR gastrointestinal OR gastric OR stomach OR esophageal OR esophagus OR gut OR intestinal OR colorectal OR colonic OR rectal OR colon OR rectum) AND (p27KIP1 OR KIP1 OR MEN4 OR MEN1B p27 OR CDKN4 OR “cyclin-dependent kinase inhibitor 1B” OR “cyclin dependent kinase inhibitor 1B” OR CDKN1B).

Relevant publcations were enrolled based on the following inclusion criteria: (1) patients were diagnosed with esophageal, gastric or colorectal cancer pathologically; (2) studies elaborated the relationships of p27 expression with prognoses or clinicopathological parameters; (3) studies were written in English or Chinese as full papers; (4) published data must be sufficient to determine the HR, OR and 95% CI; and (5) the most complete and recent article was selected when multiple articles based on the same patient set were found.

Papers were not considered based on the following exclusion criteria: (1) conference abstracts, case reports and reviews; (2) animal tests; and (3) records lacking sufficient data to calculate HR, OR and 95% CI.

Main characteristics of all of the included studies were extracted by two investigators (Dan-dan Xiong and Ai-hua Lan) independently to ensure homogeneity in information gathering and entry. The following information were gathered: first author, publication year, cancer type, cut-off value, country, sample size, follow-up period, statistical method, HR with corresponding 95% CI and study quality score. Each discrepancy was determined through discussion with a third reviewer (Gang Chen). The Newcastle-Ottawa quality assessment scale was utilized to estimate the quality of each study [85]. The HRs and 95% CIs were collected directly if they were explicitly provided in the original articles; otherwise, they were extracted from the Kaplan–Meier survival curves using Engauge Digitizer Version 4.1. Multivariate analysis can better reveal the influence of multiple factors on the survival response. Therefore, the multivariate HRs and 95% CIs were selected if multivariate and univariate analyses were available in the same study. Then we calculated the pooled HR to estimate the prognostic value of p27 in patients with DTCs. Furthermore, stratified analyses according to cancer types, statistic methods and cut-off values were performed to further analyze the associations between p27 expression and prognoses in different subgroups. We divided the studies into multivariate analysis, univariate analysis or survival curve group by statistic methods and esophageal cancer, gastric cancer or colorectal cancer group according to cancer types. We also classified the studies into high or low cut-off group with a ‘‘cut-off’’ value of 0.25, which were employed to determine p27 as low or high staining by Zhu et al [56] and Zhong et al [62], respectively. The ORs with 95% CIs were applied to investigate the relationships between p27 expression and lymph node metastasis (negative *vs.* positive), distant metastasis (negative *vs.* positive) and pathology grading (G1+G2 *vs.* G3+G4). A HR>1 implied a worse survival for DTCs patients in the low p27 expression group, and an OR>1 indicated that a decreased p27 expression was positively correlated with lymph node metastasis, distant metastasis or poor tumor differentiation. The corresponding 95% CI for the pooled HR/OR did not overlap 1 was also required. The Cochrane Q test (i.e., the chi-squared test; χ2) and the Higgins I-squared test **(I**^2^**)** were used to detect potential heterogeneity among the included studies. If I^2^ < 50% or *P* > 0.05, a fixed-effects model was used; otherwise, a random-effects model was applied. Publication bias was relevant only if there were at least 10 related studies. Otherwise, this model was underpowered and would result in unjustified conclusions [86]. Begg’s and Eeggr’s tests were applied to test publication bias when the number of relevant studies was greater than 10. All meta-analyses were carried out on STATA12.0 (Stata Corporation, College Station, TX, USA), and *P* < 0.05 was considered statistically significant.

### Data acquisition from GEO, arrayexpress, oncomine and TCGA

DTCs-related mRNA microarray and RNA-seq datasets were obtained from GEO (http://www.ncbi.nlm.nih.gov/geo/), ArrayExpress (http://www.ebi.ac.uk/arrayexpress/) and Oncomine (https://www.oncomine.org/resource/main.html) up to August 5, 2017. Search terms were ((digestive OR gastrointestinal OR gastric OR stomach OR esophageal OR esophagus OR gut OR intestinal OR colorectal OR colonic OR rectal OR colon OR rectum) AND (malignan* OR cancer OR tumor OR tumour OR neoplas* OR carcinoma OR adenocarcinoma) AND (gene OR mRNA)). Additionally, the updated RNA-seq gene profiles and corresponding clinical information of DTCs were also downloaded from TCGA data portal (http://cancergenome.nih.gov/).

Following inclusion principles were used to screen eligible datasets: (1) the research objects were human beings and the participants within in cancer group were diagnosed with esophageal, gastric or colorectal cancer pathologically; (2) the number of samples included in each dataset containing p27 gene expression value in cancer group and normal controls was not less than three; (4) the expression data of p27 gene in each group was provided and could be further analyzed.

Two investigators (Dan-dan Xiong and Rong-quan He) collected essential characteristics of each included dataset independently, with disagreements were settled through discussion with a third investigator (Gang Chen). Following details were retrieved: first author, publication year, region, cancer type, sample type, data source, platform, sample size, and expression values of p27 gene in both cancer and control groups.

All expression values of p27 gene were log2-transformed. The overall SMD was calculated and the SROC analysis was conducted to explore the expression level and diagnostic performance of gene p27 in DTCs based on all of the included datasets using STATA 12.0 (Stata Corporation, College Station, TX, USA). Independent-samples *t*-test, Log-Rank test and Kaplan–Meier survival curve were performed to investigate the correlations of gene p27 expression with clinicopathological characteristics (pathology grading, T stage, N stage, M stage and TNM stage) and prognoses in patients with DTCs based on clinical data obtained from TCGA using SPSS 20.0 (IBM, New York, USA). A *P* < 0.05 was considered to be statistical significance.

## SUPPLEMENTARY MATERIALS FIGURES AND TABLES







## Abbreviations

DTCs: digestive tract cancers
ESCA: esophageal cancer
ESAC: esophageal adenocarcinoma
ESCC: squamous cell carcinoma
GC: gastric cancer
CRC: colorectal cancer
TCGA: The Cancer Genome Atlas
GEO: Gene Expression Omnibus
CNKI: China National Knowledge Infrastructure
NR: data not reported
IHC: immunohistochemistry
OS: overall survival
DFS: disease-free survival
CSS: cancer-specific survival
HR: hazard ratio
OR: odds Ratio
SMD: standard mean deviation
CI: confidence interval
SROC: summary receiver operating characteristic
AUC: area under the curve
