# Supplementary material for: Clinical significances of p27 in digestive tract cancers: a comprehensive analysis on immunohistochemistry staining, published literatures, microarray and RNA-seq data
Source: Oncotarget. 2018 Jan 23;9(15):12284–303. doi: 10.18632/oncotarget.24316 (PMC5844746; doi:10.18632/oncotarget.24316)
Supplement: Supplementary file 1 [file oncotarget-09-12284-s001.pdf]

Clinical significances of p27 in digestive tract cancers: a comprehensive analysis on immunohistochemistry staining, published literatures, microarray and RNA-seq data

SUPPLEMENTARY MATERIALS

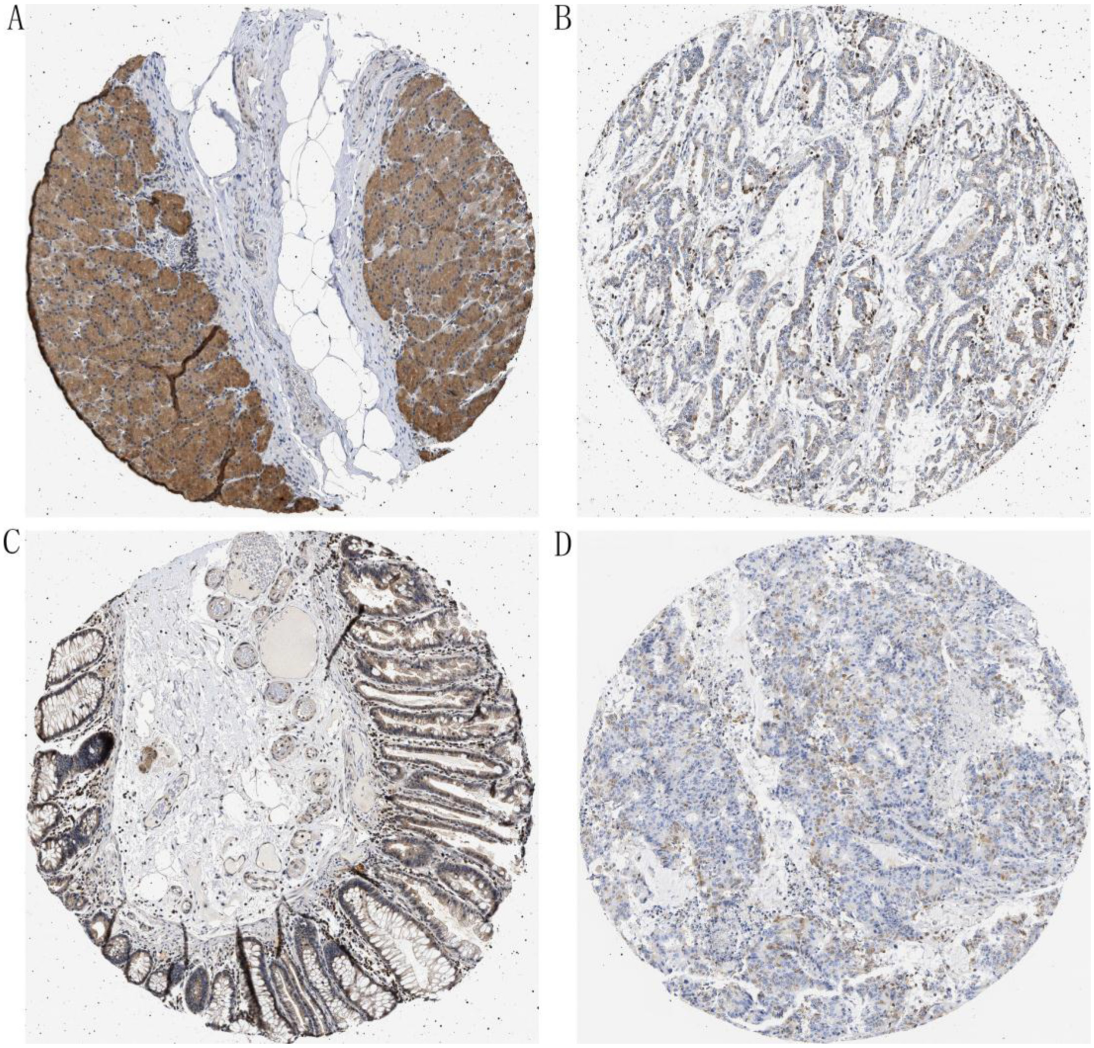

Supplementary Figure 1: Immunohistochemistry staining of p27 protein based on the human protein atlas database. (**A**) A paracancerous gastric tissue showed a high p27 protein expression; (**B**) A gastric adenocarcinoma tissue showed a low p27 protein expression; (**C**) A colorectal tissue showed a high p27 protein expression; (**D**) A colorectal cancer tissue showed a low p27 protein expression. Magnification: ×100.

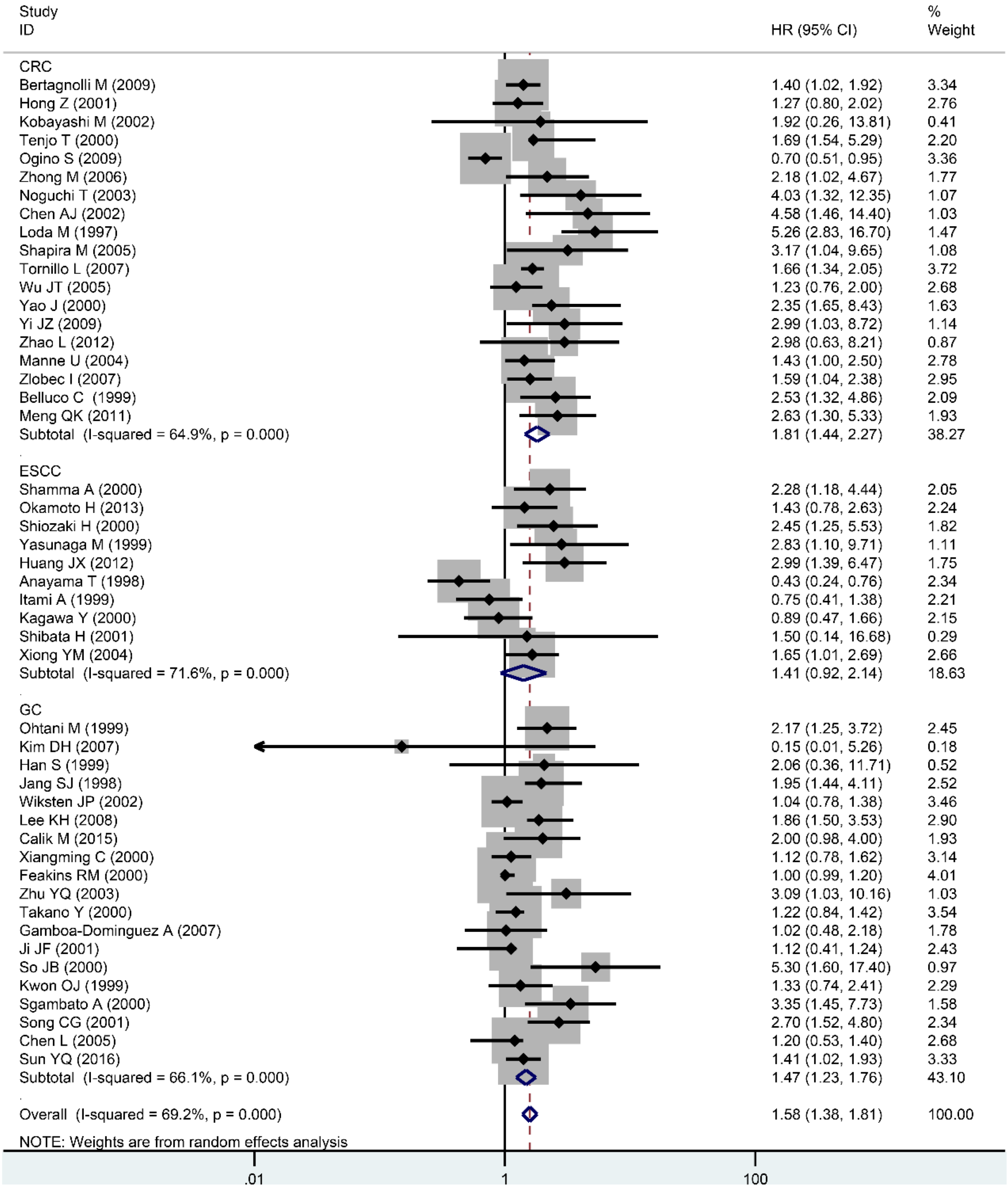

Supplementary Figure 2: Forest plot of the pooled HR for OS. Result of subgroup analysis on cancer types.

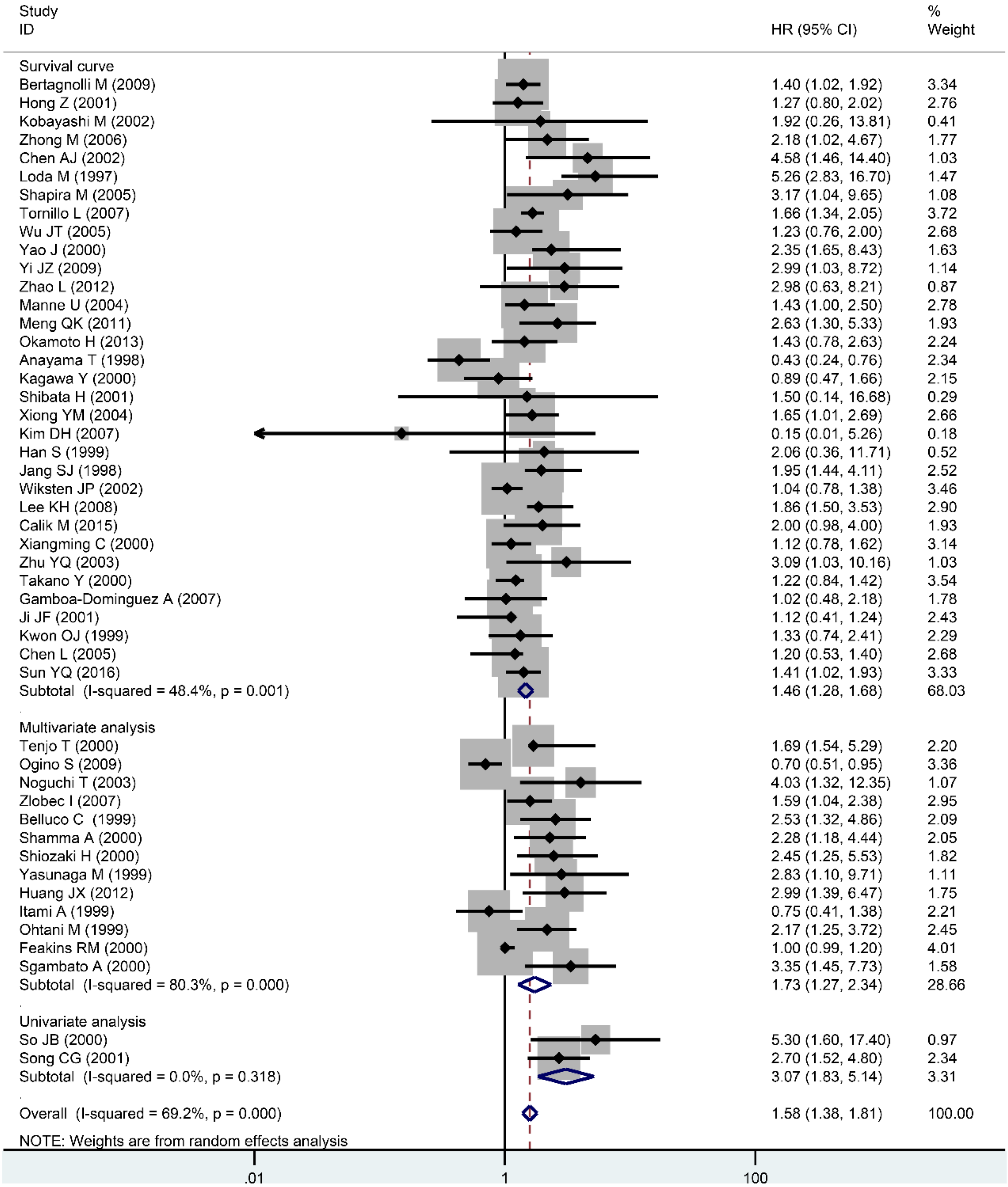

Supplementary Figure 3: Forest plot of the pooled HR for OS. Result of subgroup analysis on statistical methods.

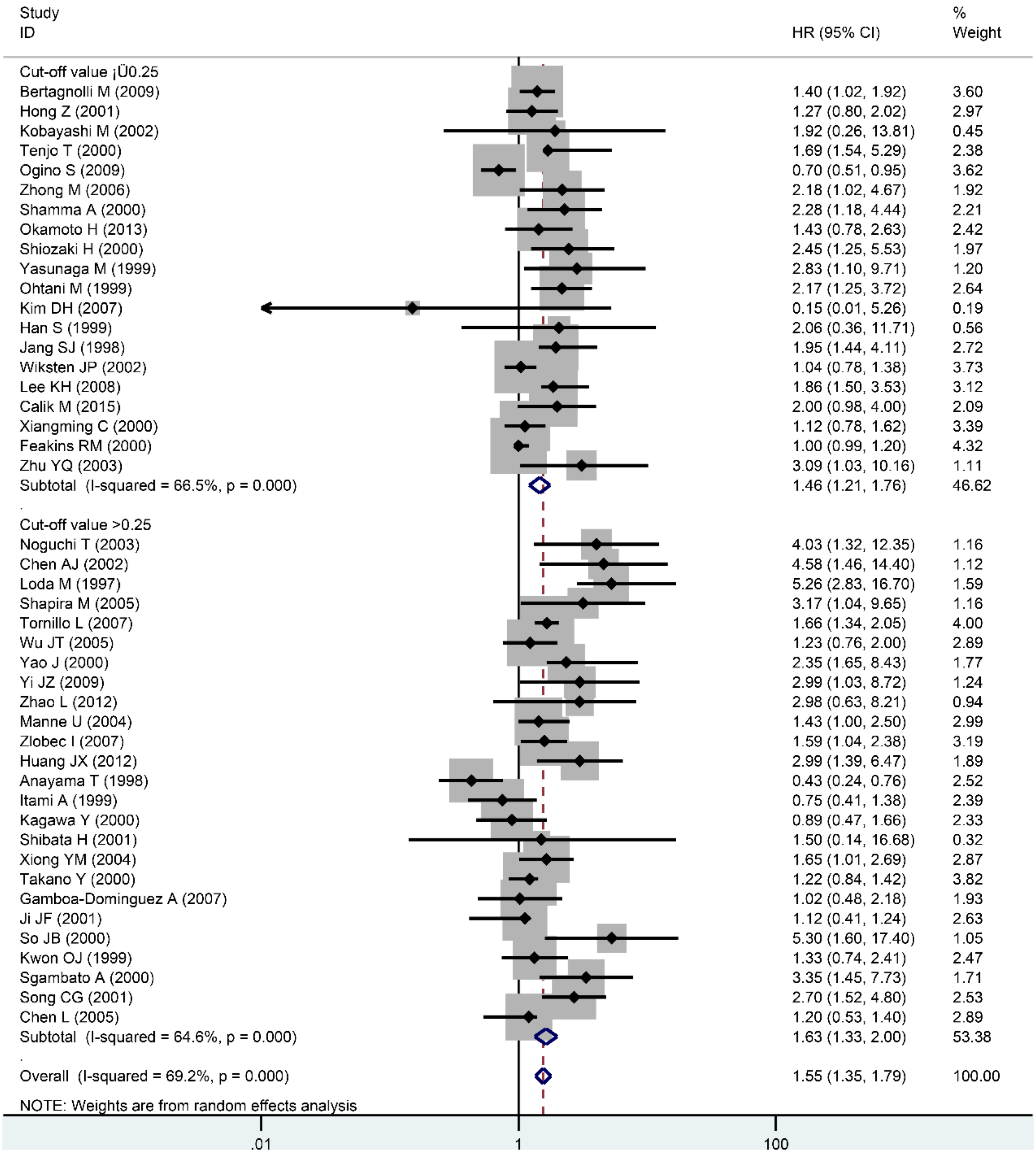

Supplementary Figure 4: Forest plot of the pooled HR for OS. Result of subgroup analysis on cut-off values.

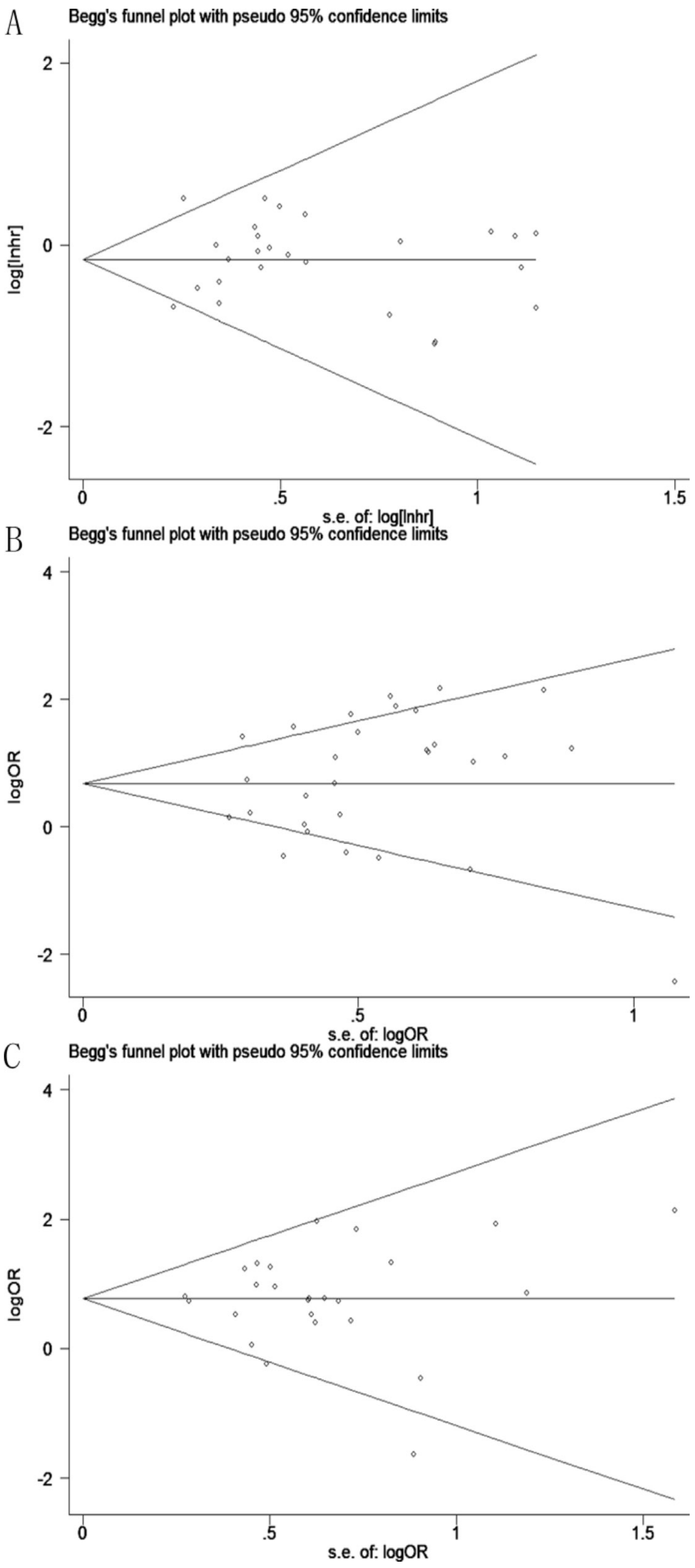

Supplementary Figure 5: Potential publication bias among the included studies. (**A**) Funnel plot of the 48 included studies for OS. (**B**) Funnel plot of the 29 included studies for lymph node metastasis. (**C**) Funnel plot of the 25 included studies for pathology grading.

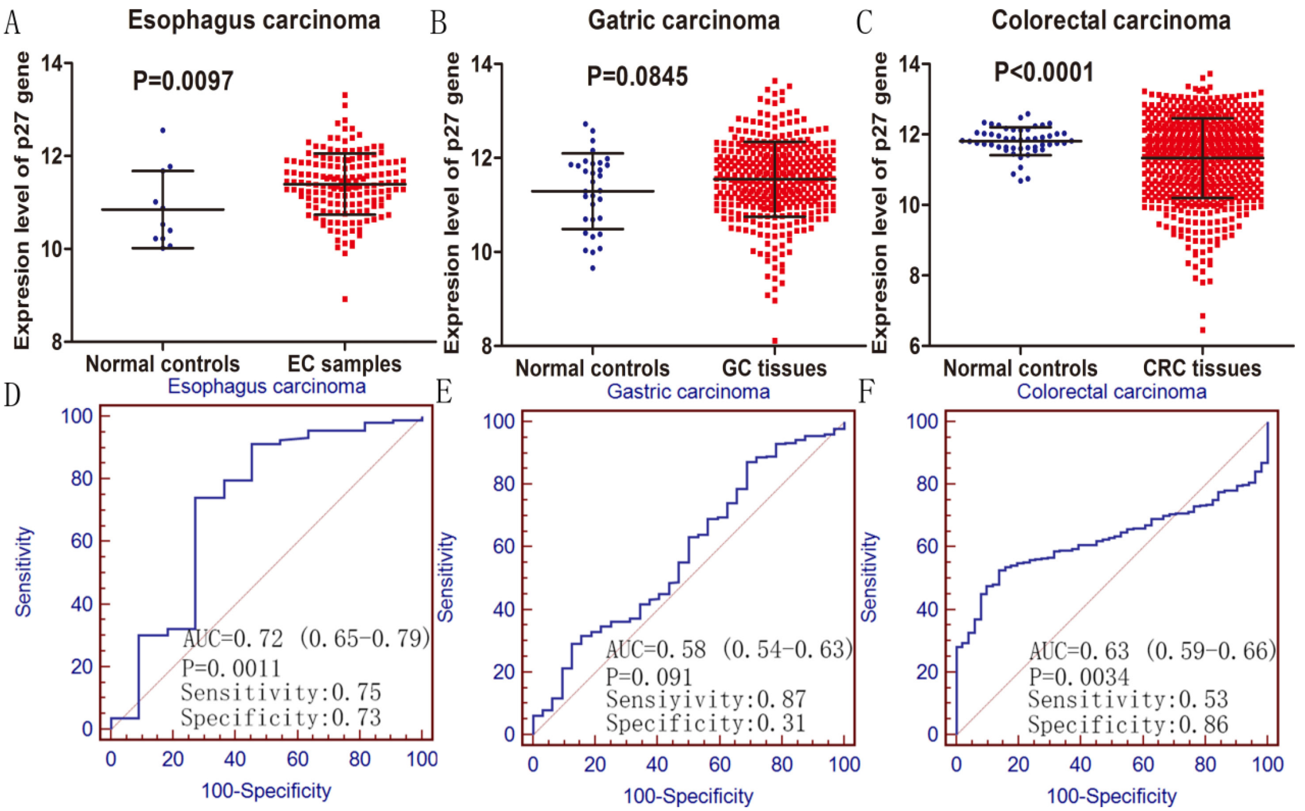

Supplementary Figure 6: Expression level and diagnostic value of p27 gene in esophagus carcinoma (**A** and **D**), gastric carcinoma (**B** and **E**) and colorectal carcinoma (**C** and **F**) based on data from TCGA.

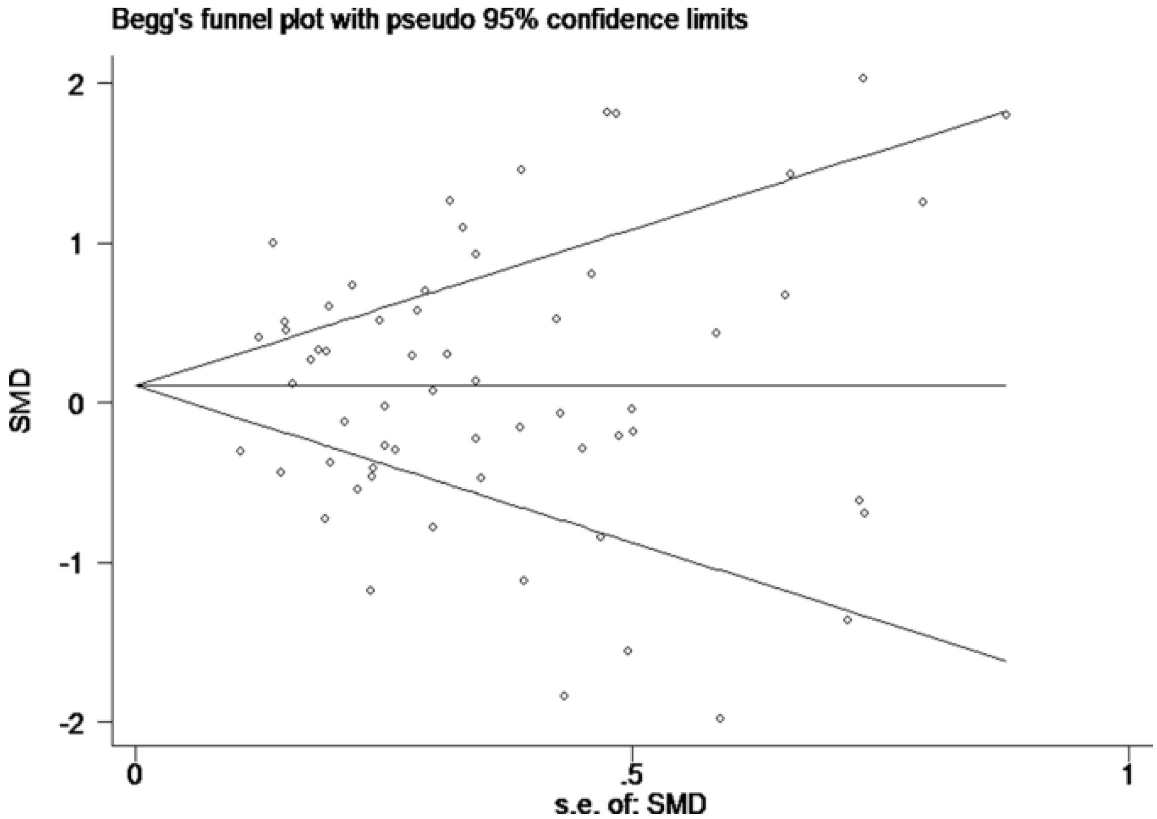

Supplementary Figure 7: Funnel plot evaluating potential publication bias among the 60 included studies.

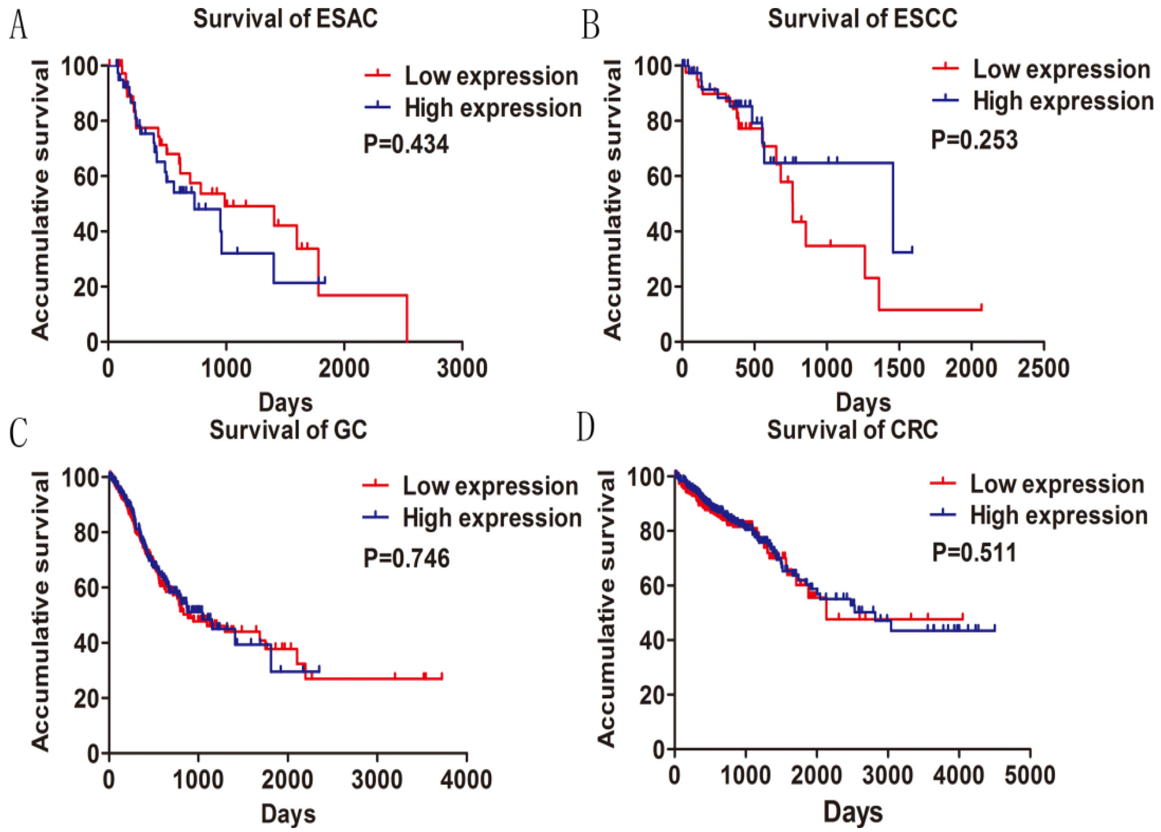

Supplementary Figure 8: Kaplan–Meier survival curves evaluating prognostic value of p27 gene in DTCs based on data from TCGA. (**A**) Prognostic value of p27 gene in ESAC; (**B**) Prognostic value of p27 gene in ESCC; (**C**) Prognostic value of p27 gene in GC; (**D**) Prognostic value of p27 gene in CRC.

Supplementary Table 1: Relationships between p27 gene expression and clinicopathological parameters in DTCs based on data from TCGA

Cancer type	Clinicopathological parameters	Group	Number of patients	Mean ± SD	T value	*P* value
**ESCC**	**Pathology grading**	G1 + G2	52	11.38 ± 0.73	0.351	0.727
		G3 + G4	19	11.31 ± 0.82		
	**T stage**	T1 + T2	34	11.22 ± 0.85	0.969	0.336
		T3 + T4	44	11.39 ± 0.65		
	**N stage**	N0	45	11.30 ± 0.90	0.282	0.778
		N1 + N2	32	11.35 ± 0.47		
	**M stage**	M0	70	11.35 ± 0.74	0.684	0.496
		M1	3	11.05 ± 0.66		
	**TNM stage**	Stage I + II	53	11.28 ± 0.81	0.746	0.458
		Stage III + IV	25	11.41 ± 0.57		
**ESAC**	**Pathology grading**	G1 + G2	30	11.36 ± 0.54	1.205	0.234
		G3 + G4	25	11.56 ± 0.64		
	**T stage**	T1 + T2	31	11.44 ± 0.44	0.059	0.953
		T3 + T4	36	11.45 ± 0.64		
	**N stage**	N0	21	11.24 ± 0.42	2.009	0.069
		N1 + N2	46	11.53 ± 0.59		
	**M stage**	M0	51	11.43 ± 0.55	0.985	0.329
		M1	5	11.68 ± 0.28		
	**TNM stage**	Stage I + II	3	11.37 ± 0.42	1.528	0.132
		Stage III + IV	32	11.57 ± 0.62		
**GC**	**Pathology grading**	G1 + G2	147	11.44 ± 0.76	1.856	0.064
		G3 + G4	219	11.60 ± 0.82		
	**T stage**	T1 + T2	99	11.52 ± 0.75	0.521	0.603
		T3 + T4	268	11.57 ± 0.82		
	**N stage**	N0	11	11.59 ± 0.74	0.603	0.547
		N1 + N2	247	11.54 ± 0.83		
	**M stage**	M0	330	11.57 ± 0.79	2.279	0.053
		M1	25	11.19 ± 0.88		
	**TNM stage**	Stage I + II	164	11.56 ± 0.74	0.037	0.97
		Stage III + IV	190	11.56 ± 0.86		
**CRC**	**T**	T1 + T2	127	11.16 ± 1.07	1.794	0.073
		T3 + T4	494	11.36 ± 1.14		
	**N**	N0	253	11.35 ± 1.13	0.738	0.461
		N1 + N2	266	11.28 ± 1.13		
	**M**	M0	460	11.25 ± 1.16	0.577	0.564
		M1	87	11.18 ± 1.03		
	**Stage**	Stage I + II	335	11.34 ± 1.13	0.768	0.443
		Stage III + IV	268	11.27 ± 1.14		

Supplementary Table 2: The essential characteristics and quality scores of the 62 published literatures included in the meta-analysis. See_Supplementary_Table 2

Supplementary Table 3: The main information of the 60 microarray and RNA-seq datasets included in the meta-analysis. See_Supplementary_Table 3
